# Improving survival and efficacy of pluripotent stem cell–derived cardiac grafts

**DOI:** 10.1111/jcmm.12147

**Published:** 2013-10-09

**Authors:** Creighton W Don, Charles E Murry

**Affiliations:** 1 Department of Medicine/Cardiology, University of WashingtonSeattle, WA, USA; 2 Departments of Pathology and Bioengineering, Center for Cardiovascular Biology, Institute for Stem Cell and Regenerative Medicine, University of WashingtonSeattle, WA, USA

**Keywords:** cardiac, cardiomyocyte, myocardium, pluripotent stem cell, human embryonic stem cell, differentiation, apoptosis, homing, tissue engineering, hydrogel

## Abstract

Human embryonic stem cells (hESCs) can be differentiated into structurally and electrically functional myocardial tissue and have the potential to regenerate large regions of infarcted myocardium. One of the key challenges that needs to be addressed towards full-scale clinical application of hESCs is enhancing survival of the transplanted cells within ischaemic or scarred, avascular host tissue. Shortly after transplantation, most hESCs are lost as a result of multiple mechanical, cellular and host factors, and a large proportion of the remaining cells undergo apoptosis or necrosis shortly thereafter, as a result of loss of adhesion-related signals, ischaemia, inflammation or immunological rejection. Blocking the apoptotic signalling pathways of the cells, using pro-survival cocktails, conditioning hESCs prior to transplant, promoting angiogenesis, immunosuppressing the host and using of bioengineered matrices are among the emerging techniques that have been shown to optimize cell survival. This review presents an overview of the current strategies for optimizing cell and host tissue to improve the survival and efficacy of cardiac cells derived from pluripotent stem cells.

- Introduction
- Cell differentiation, selection and protection
  – Impact of cell differentiation and selection
  – Improving cell survival: conditioning the cells
- Optimizing host myocardium
  – Modifying Inflammation and Immunogenicity
  – Angiogenesis
  – Extracellular and bioengineering solutions
- Conclusions and future challenges of functional integration

## Introduction

Over the past decade, significant advances have been made in generating viable cardiac cells from pluripotent stem cells. Several protocols have been developed to guide the differentiation of human embryonic stem cells (hESC) and induced pluripotent stem cells (iPSCs) into functioning cardiomyocytes 1,2, and successfully engraft and integrate these cells into pig and rodent hearts 1,3. Nevertheless, there remain several challenges for maintaining cell survival and function after transplant that will need to be overcome to regenerate cardiac tissue that will substantially improve heart function.

Following transplant, a high rate of stem cell loss is observed, as a result of multiple mechanical, cellular and host factors. Several studies have demonstrated that only 10–30% of stem cells remain within a few days of transplantation, with that number decreasing to 5–15% over 10–12 weeks 4–5. Mechanical factors may play a large role as cells are extruded from the injection sites by the squeezing action of beating hearts 6. Studies of radiolabelled cells 7 and microspheres 8 in a porcine infarct model demonstrated that less than 10% end up in the heart immediately following direct injection, with even lower retention for intracoronary and intravenous delivery. Most of the labelled cells and microspheres were found in the lung shortly after injection, indicating that the cells enter damaged cardiac veins and leave through the coronary venous effluent. Of cells that are retained, a high proportion undergo apoptosis or necrosis 9, probably because of loss of adhesion-related signals, ischaemia, inflammation and, depending on the context, immunological rejection. Most of the transplanted cells that are retained after the initial injection die in the first few days, with DNA fragmentation stains showing cell death indices as high as 90% 9. Blocking the apoptotic signalling pathways of the cells along with modifying an otherwise inhospitable host environment characterized by hypoxic and inflamed infarcted tissue may be keys to improving cell survival. This review presents an overview of the current strategies for selecting and conditioning cells, providing pro-survival cocktails, optimizing host tissue conditions and providing hydrogel delivery systems to improve the survival and efficacy of cardiac cells derived from pluripotent stem cells (Table 1). We focus on hESC derivatives when possible, but include other cell types where appropriate.

**Table 1:** Strategies described to improve cardiac embryonic stem cell survival

Cellular protection
*Cell conditioning*
Heat shock
Hypoxic pre-conditioning
Hypoxia inducible factor-1
Diazoxide
Isoflurane
Erythropoietin
*Anti-apoptotic pathways*
Rho-associated kinase inhibition
TGF-β_2_ treatment
SDF-1 signalling of PI3K/Akt
p38 MAPK inhibition
*Pro-survival cocktail*
Akt and Bcl overexpression
Pinacidil
Cyclosporine
ZVAD-fmk
Insulin-like growth factor-1
Optimizing host myocardium
*Inflammation inhibition*
CD4/CD8/C3 inhibition
Prednisone
Cyclosporine
*Angiogenic and homing factors*
FGF
VGEF
SDF-1a/CXCR4
*Co-transplant*
Mesenchymal cells
Fibroblasts
Endothelial cell progenitors
Improving retention
*Hydrogels and bioengineering solutions*
Collagen
Hyaluronic acid
Matrigel
Fibrin
Chitosan
Oligopolyethylene
Alginate
Magnetic targeting
Engineered cell sheets

## Cell differentiation, selection and protection

### Impact of cell differentiation and selection

Several investigators have described successful production of definitive, beating cardiomyocytes from human embryonic stem cells 1–10 and, more recently, from induced pluripotent stem cells (iPSCs) 11. While early reports used relatively inefficient methods such as dissection of beating regions from embryoid bodies or undefined induction conditions such as co-culture with endodermal cells, several robust protocols now exist using defined signalling molecules to direct the differentiation of pluripotent cells to mesoderm and on to cardiomyocytes 1–10. These protocols typically yield cardiomyocyte purities of >50% without additional purification. While there is a genuine concern for transferring undifferentiated cells, which can form teratomas and non-cardiac cells, there is an equally important thought that the survival of the cardiac cells depends on the co-transfer of supporting stromal cells, such as fibroblasts 12 and endothelial cells 13. Among the cardiac cells produced, there are also varying proportions of atrial, ventricular and nodal phenotype, depending on the differentiation protocol used. The type of cardiac cell generated is of key importance, not only for replacing the diseased tissue of interest (producing ventricular cells to treat a myocardial infarction, for instance), but to optimally co-ordinate the mechanical and electrical integration of the grafted cells with surrounding tissue 14, as there are unique differences in the action potentials of the different cell types. A mixed population of cardiomyocytes and stromal cells derived from hESCs has been used successfully in animal studies 4, and has been shown to produce extracellular matrix that may be important for survival of the graft 13. Therefore, effective hESC therapy will require tailoring the differentiation and selection of cardiomyocytes to the clinical scenario, as well as determining the proper mix of co-transplanted cells to enhance integration and survival of the graft.

### Improving cell survival: conditioning the cells

Once cardiomyocytes have been derived from hESCs, two principal strategies have been explored to improve their survival in infarcted myocardium: pre-treating cells to induce endogenous cellular survival mechanisms and using chemical or biologic inhibitors of major cell death pathways 15. A third strategy, optimizing the host tissue to receive the graft, is considered in the next section.

**Figure 1:**
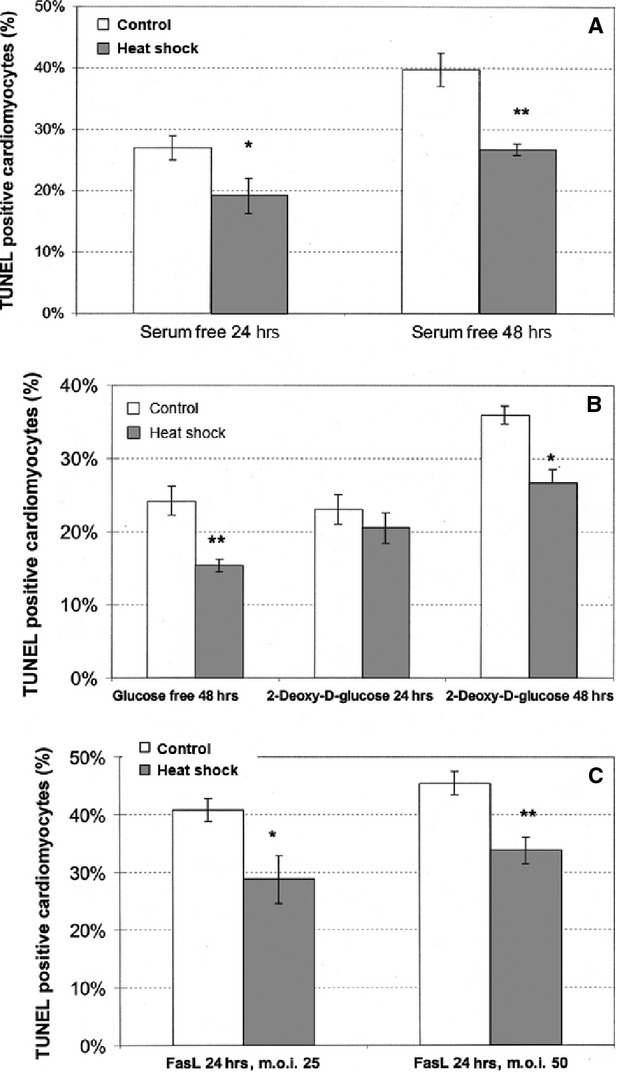
Heat shock improves cardiomyocyte survival. Heat shock protects cardiomyocytes from death stimuli *in vitro*. Neonatal cardiomyocytes were heat shocked at 43°C for 45 min. and subjected to death stimuli 1 day later. TUNEL staining was quantified only in cardiomyocytes, identified by myosin heavy chain double staining. Heat-shocked cardiomyocytes showed a significant reduction in TUNEL staining 24 or 48 hrs after the death stimuli. (A) Serum deprivation. (B) Glucose deprivation with or without 1 mmol/l 2-deoxy- -glucose. (C) Fas ligand (FasL) adenoviral infection at 25 or 50 particles/cell. Results are mean from three replicate wells, and were reproduced in two to three separate isolates of cardiomyocytes. ∗∗*P* < 0.01, ∗*P* < 0.05. From Ref. 9.

**Figure 2:**
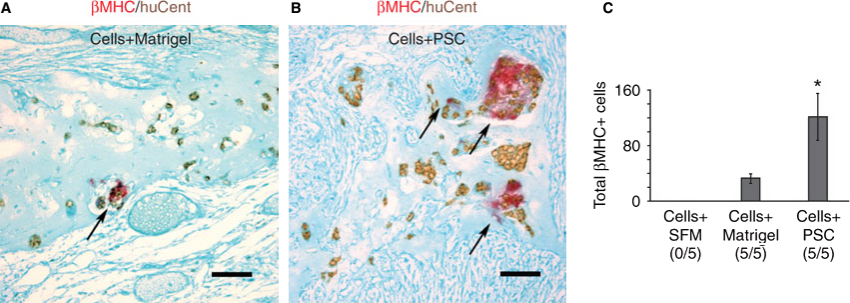
Histological analysis of graft cell survival with Matrigel and pro-survival factors. Histological analysis of graft cell survival. Heat-shocked hES cell–derived cardiomyocytes were injected into infarcted hearts of nude rats in the presence of SFM, Matrigel-only (A, Cells+Matrigel) or the full pro-survival cocktail (PSC) including Matrigel (B, Cells+PSC) (*n* = 5 per group). Sections were stained with an antibody to β-myosin heavy chain (β-MHC, red chromagen) as well as a human-specific pan-centromeric *in situ* hybridization probe (huCent, brown DAB deposit) to identify total human (that is, huCent+) and, specifically, human cardiac (that is, β-MHC and huCent double-positive) graft cells. The human cardiomyocytes, indicated by arrows, were significantly more abundant in histological sections from the Cells+PSC group than in Cells+Matrigel alone group. Histology is not depicted from the recipients of cells in SFM alone because none of these hearts showed even a single surviving human nucleus after 1 week. Counterstain, fast green; scale bar, 50 μm. (C) Quantification of hES cell–derived cardiomyocyte graft size. Although no grafts were detected in any rats receiving hES cell–derived cardiomyocytes delivered in SFM alone (Cells+SFM), all rats receiving cells delivered in Matrigel-only (Cells+Matrigel) or in the full pro-survival cocktail (Cells+PSC) showed surviving graft (5/5 rats per group). However, recipients of cells in the full pro-survival cocktail (Cells+PSC) showed a mean of approximately fourfold more β-myosin–positive graft cells than did the Matrigel-only group. Note that counts indicate the total number of cells observed on sampled sections, not the total number of cells per heart. *P < 0.05. From Ref. 1.

Heat shocking of cells (*e.g*. 43°C for 30 min.) induces a highly conserved transcriptional pathway that results in synthesis of cytoprotective proteins, including heat shock proteins such as Hsp60, Hsp70 and Hsp90, as well as antioxidants. Heat-shocked rat and hESC-derived cardiomyocytes have increased survival following exposure to death stimuli *in vitro* (Figs 1 and 2). Importantly, when cells were grafted into a rat heart infarct, heat shock reduced cell death by half on the first day and resulted in threefold larger graft size at 1 week 9. Similarly, adaptive responses to hypoxia can have protective effect on cells through up-regulation of hypoxia-inducible factor (HIF-1) that activates several pathways promoting cell proliferation, angiogenesis and survival within ischaemic, low-oxygen microenvironments. hESCs cultured in a 3% oxygen suspension produce highly angiogenic embryoid bodies, marked by increased expression of VEGF receptors and the emergence of endothelial cells 16. Hypoxic pre-conditioning of cardiomyocytes could potentially help these cells better withstand the ischaemic environment of an acute myocardial infarction or poorly vascularized scar tissue, as well as increase the population of cells with a vascular fate co-transplanted with cardiomyocytes. Drugs that open mitochondrial ATP-dependent potassium channels, such as diazoxide and isoflurane, have been widely demonstrated to protect cardiomyocytes from ischaemic injury 17. Investigators have demonstrated similar improvement in survival after pre-treating skeletal myoblasts with these drugs prior to transplantation in a myocardial infarction model 18. Transfecting stem cells to overexpress VEGF 19 or co-administering myoblasts with adenovirus-encoded HIF-1 20 have had promising results in terms of cell survival and engraftment, although these pathways will need to be turned off once a desired vascular density is achieved. Hypoxia has also been shown to induce expression of chemokine receptor-4 CXCR4 (which binds to stromal-derived growth factor SDF-1) in murine cardiac progenitor cells, which can promote homing and engraftment to ischaemic myocardium 21. More recently, investigators have demonstrated enhanced survival of hESCs with Rho-associated kinase inhibition 22, transforming growth factor (TGF) -β_2_ treatment 23, p38MAPK inhibition 24 and a novel pathway involving SDF-1 signalling of PI3K/Akt 25. The relative efficacy or synergistic benefits of blocking these additional pathways have yet to be explored.

Directly stimulating anti-apoptotic pathways in hESCs and their derivatives has been reviewed previously 1,9. Phosphoinositide 3-kinase (PI3K) regulates translocation of serine-threonine kinase Akt that in turn mediates several signalling pathways involved in cellular proliferation and survival, and inhibition of apoptosis. Transgenic overexpression of Akt can improve survival of some populations of transplanted cells 9, but studies of hESC-derived cardiomyocytes showed no benefit when adenoviral Akt was used as a single survival strategy 9, possibly as a result of cell death caused by the adenoviral infection. Overexpression of Bcl-2, another anti-apoptotic protein, and treatment with insulin-like growth factor (IGF-1)—which stimulates Akt, had similarly unfavourable results for hESC-derived cardiomyocytes 1, despite showing improvement in cardiac cell survival in other cell lines 26. Use of the caspase inhibitor ZVAD also failed to improve cell survival 5. On the other hand, incubating hESC-derived cardiomyocytes with carbamylated erythropoietin, which initiates Akt phosphorylation, significantly increased graft survival when combined with heat shock 15.

One of the main lessons our group learnt was that there are many pathways through which cardiomyocytes can die after transplantation, and that blocking a single one is typically insufficient to enhance graft size. For example, when attempting to enhance the size of hESC-derived cardiomyocyte grafts, we tested a large number of anti-necrotic and anti-apoptotic interventions, designed to activate or inhibit a single pathway. These included viral overexpression of Akt (a pro-survival kinase) or Bcl2 (a blocker of mitochondrially induced death), treatment with the pro-survival cytokine IGF-1, blocking ‘danger signals’ from nucleic acid breakdown with allopurinol and uricase, natural killer cell depletion and broad-spectrum immunosuppression. None of these individual interventions prevented graft cell death. However, when we combined heat shock with a ‘pro-survival cocktail’ made up of Matrigel, IGF-1, a Bcl X_L_-derived peptide, pinacidil (which opens mitoK_ATP_), cyclosporine (at a sub-immunosuppressive dose that acts principally on the mitochondrial permeability transition pore) and the caspase inhibitor ZVAD-fmk, we observed greatly improved engraftment and survival 4 weeks post-transplant compared with cells transplanted with Matrigel only or cells delivered in serum-free medium (which had no detectable grafts; Fig. 2) 1.

## Optimizing host myocardium

Myocardium that has been acutely or chronically infarcted presents an unwelcoming environment for the survival of transplanted cardiomyocytes. The large amount of cell necrosis in acutely infarcted tissue is associated with significant quantities of inflammatory cells, reactive oxygen species and cytokines that are toxic to the grafted cell, while the scar tissue of an old infarction can be poorly receptive to the engraftment and integration of new cells. In both situations, the ischaemia associated with the non-vascularized cell grafts presents an immediate challenge to cell survival, and the positive outcome of blocking cell death and augmenting cell survival pathways speaks to the need to overcome the inhospitable ischaemic and inflammatory environment.

### Modifying inflammation and immunogenicity

The inflammatory cytokine interleukin (IL)-1 is elevated in acutely infarcted myocardium, and mediates cardiac myocyte apoptosis and adverse remodelling 27. Skeletal myoblasts that express an IL-1 inhibitor had improved survival after transplantation into infarcted myocardium than control cells, by over sixfold at 3 weeks associated with improved left ventricular size and function and reduced fibrosis 28. Co-injection of skeletal myoblasts with superoxide dismutase into an acute infarct reduced levels of tumour necrosis factor-α, TGF-β, IL-1β, IL-6, GM-CSF, while improving cell survival 29.

In contrast to autotransplant studies with adult stem cells or circulating progenitor cells, allograft transplant of hESCs elicits an adaptive immunological response and compounds the problem of cells trying to survive in this inflammatory milieu 30. Given the inherent incompatibilities of xenotransplantation, animal studies evaluating hESC-derived cardiomyocyte grafts have used immunologically compromised hosts, such as athymic rats 1 or immunodeficient mice 4. In studies of allogenic donor skeletal myoblasts transplanted into the ischaemic hind limb of mice, pre-treatment of the host tissue with antibodies against CD4, CD8 and C3 complement increased survival of the graft 31. Treatment of host tissue with prednisone and cyclosporine has been used successfully to transplant hESC into immunocompetent mice 3. In clinical use, down-regulating host immunity prior and subsequent to transplant will be required until means of inducing tolerance (*e.g*. through haematopoietic chimerism) 32 or immune-evading grafts are generated. Several studies indicate that ESCs have low or undetectable levels of class I or II major histocompatibility antigens, but after differentiation, both class I and II antigens can be up-regulated by inflammatory cytokines 30. Thus, while immature ESC derivatives may have reduced immunogenicity compared with adult transplanted tissue, as they mature it seems likely that they will have the full immunogenicity of an adult cell 33. One encouraging possibility is that cell grafts, because of their relative simplicity compared with whole organs, may require less intense immunosuppression regimens than those used currently for organ transplantation.

### Angiogenesis

Improving blood flow to infarcted myocardium has also been explored as a means to reduce the persistent ischaemia of the host tissue that threatens the survival of grafts. Pre-treating the host myocardium with adenovirus encoding VEGF 3 weeks prior to transplanting foetal cardiomyocytes led to increased capillary density in the infarct and higher rate of survival of the transplanted cells 34. Fibroblast growth factor (FGF) given 1 week prior to foetal cardiomyocyte cell grafting into infarcted hearts was associated with enhanced ventricular size and function, and greater distribution of transplanted cells throughout the scar area 35. The improvement in ventricular function in this study may be a result of angiogenesis, or a direct effect of the growth factors on the cells, but nevertheless has positive implications for maximizing cardiac regeneration. Another approach to improving blood supply is to co-transplant hESC-derived endothelial cells along with hESC-derived cardiomyocytes, to promote neovascularization of the graft 13.

### Extracellular and bioengineering solutions

While direct intramyocardial injection of cells results in a greater retention of cells than intracoronary or intravenous methods, as much as 90% of the cells can be immediately lost from the injection site because of mechanical extrusion from the injection track and washout of cells into the circulation 6,7. Furthermore, when cell attachments to extracellular matrix or other cells are lost, as in during transfer of cardiomyocytes from cell culture into the host myocardium, an apoptotic pathway is initiated, termed anoikis. The loss of adhesion-related survival signals will eventually lead to cell death unless those attachments are re-established. For these reasons, the use of biomaterials has been explored as a ways of mechanically increasing retention of cells and mimicking extracellular matrix, as well as provide a niche environment with a depot of pro-survival factors and drugs. These agents have generally been hydrogels composed of synthetic polymers or natural proteins 36–48.

The most widely used hydrogels have properties resembling extracellular matrix that can provide adhesive peptides to maintain survival signalling of transplanted hESCs 43. *In vitro*, hESCs cultured and transplanted in collagen have reduced apoptosis 40. Furthermore, collagen patches provide a three-dimensional framework into which transplanted embryonic stem cells can align 39. Matrigel is a gelatinous biologic mixture that has also been used successfully in delivering hESC, which, when combined with pro-survival factors, has been associated with improved survival and engraftment of hESC cardiomyocytes into infarcted tissue 1. The mixture itself manifests angiogenic properties when injected into infarcted myocardium and may promote survival through multiple pathways 44.

Fibrin glue is generated by mixing fibrinogen and thrombin and has been successfully used in the injection track to prevent extrusion of skeletal myoblasts transplanted into a myocardial infarction, resulting in a greater number of cells in the target site, smaller scar and increased arteriole density 36. Bioengineered hydrogels can be designed to be pH- or temperature-sensitive, hence they take the form of an injectable liquid for cell suspension, which converts into a biodegradable gel with adhesive properties within heart tissue 38. A chitosan hydrogel with such properties has been used to deliver embryonic stem cells in a rat myocardial infarction model 42. *In vitro*, cells survived, proliferated and aggregated, and following injection into the infarct, the chitosan formed a temporary scaffold that improved cell retention and survival, resulting in a significantly larger graft compared with direct cell injections alone. A study performed with Rat H9c2 neonatal heart cells injected into infarcted myocardium with a mixture of collagen gel and Matrigel had a threefold increase in survival compared with direct cell injections or either vehicle alone (Fig. 2) 40. Hyaluronic acid–based gels are also appealing for co-injection, as this glycosaminoglycan is a component of the naturally occurring extracellular matrix found within connective tissues. It has been shown to improve wall thickness and angiogenesis in infarcted hearts 46–50. A combination of hyaluronic acid, to improve cell retention and survival, covalently linked to thiolated collagen, to aid cell attachment, demonstrated a marked improvement in retention of cardiosphere-derived cells in a mouse infarct model. This was associated with reduction in apoptosis, increased angiogenesis and with an improvement in left-ventricular function 50.

The hydrogels themselves may have ameliorative effects on ischaemic myocardium by providing structural support to the heart and promoting angiogenesis. Cell-free fibrin glue injected into infarcted myocardium prevented scar expansion and wall thinning compared with control injections 37, and induced microvessel formation within the infarct 36. Increased density of arterioles and capillaries has also been reported at the site of alginate 41 and hyaluronic acid 46–50 injections, as well as decreased host cell apoptosis in these regions, leading investigators to hypothesize that these hydrogels recruit pro-angiogenic cells while favourably modulating the inflammatory microenvironment 45.

Anti-apoptotic, angiogenic and anti-inflammatory factors can be added to the hydrogels, providing a depot of pro-survival factors for controlled release. The use of VEGF- and FGF-loaded hydrogels promotes increased capillary ingrowth and angiogenesis when added to stem cell transplants 38–40. Other investigators have demonstrated increased stem cell homing and myocardial repair associated with injected hydrogels providing sustained levels of SDF-1 51 and erythropoietin 52.

Injection of a foreign substance, particularly a synthetic material, however, may not always be beneficial. Hyaluronic acid has also been shown to impair IGF-1 signalling 49, which may lead to greater apoptosis. Investigators have shown that a hydrogel composite of oligopolyethylene glycol injected with mouse ESCs increased graft size and reduced infarct size in a rat myocardial infarction model more than cells injected alone 53. On the other hand, a study of synthetic hydrogels containing FGF achieved sustained high levels of the growth factor, but also showed extensive inflammation at the interface of the tissue and gel 38. The presence of foreign material producing inflammation could potentially disrupt electrical integration of the cells or generate circuits for arrhythmias in these sites. Extracellular matrix–based components such as hyaluronic acid and collagen may prove superior in this regard and deserve further investigation.

Other novel methods for improving cell retention have been described. One such technique uses superparamagnetic microspheres to magnetize cardiac-derived cells that are then localized and retained with a magnet superimposed over target tissue 54.

## Conclusions and future challenges of functional integration

The past decade has provided promising techniques for improving the survival of hESC-derived cardiomyocyte grafts. One of the future challenges for cardiac regeneration in the coming years will be learning how to integrate graft cell conditioning, angiogenesis, anti-apoptosis, immunosuppression, use of bioengineered matrices and other emerging strategies to optimize cell survival. In addition, the complex interaction of transplanted cardiomyocytes and host tissue must also be co-ordinated in the proper temporal sequence. Formation of a rich vascular bed, modulation of inflammation and up-regulation of anti-apoptotic factors and homing cytokines need to be timed to coincide with cell transplant, when these cells are most likely to respond to these signals 55. Clinical trials of adult bone marrow stem cell treatment of myocardial infarction have borne this problem out, suggesting that cell infusion too early after infarct, into inflamed necrotic tissue, may attenuate the benefit of the transplant.

Tissue-engineered cardiac cell sheets have been successfully constructed from pluripotent stem cells and may provide a solution to the problem of cell death as a result of loss of matrix/cell attachments 56–57, but tissue engineering raises other issues with regard to cell survival and engraftment. A vascular system must be created to support more than a thin layer of cells; the transplantation would require open surgical grafting onto the epicardium; and the epicardial graft must electromechanically integrate with the host myocardium. Finally, complex mechanical processes will be required to engineer the ideal orientation and synchronous contraction of cardiomyocytes that will need to align with the cell orientation and mechanical shortening of the donor site 56.

Beyond simply achieving survival of the hESC-derived cardiomyocytes, attention needs to be given to ensuring the optimal electromechanical function of the graft and integration with the host tissue. Kehat *et al*. demonstrated that the hESC cardiomyocytes can form structural and electromechanical connections with host tissue 3. By creating an ectopic pacemaker made of spontaneously beating embryoid bodies that was responsive to adrenergic stimulation, they proved successful coupling of hESC-derived cardiac cells with mature host cells. The difference in the intrinsic rate of the host and transplanted cells, however, may pose problems with regard to arrhythmias and ventricular synchrony. Cardiomyocytes differentiated from hESCs may have atrial, nodal or ventricular phenotypes, and immature cells within the heart may create areas of heterogeneous conduction that lead to arrhythmias. Furthermore, there can be physiological differences between host and transplanted cells that can lead to functional decoupling as a result of varied responses to hormonal and adrenergic stimuli.

While great strides have been made towards the goal of regenerating cardiac tissue with pluripotent stem cells, the road to carrying out clinical human studies requires further optimization of these pro-survival techniques. We will need to achieve large-scale remuscularization of an infarct in larger animals with grafts that are integrated electrically and mechanically into host tissue, before the ultimate goal of regenerating the human heart can be realized.

## References

[b1] Laflamme MA, Chen KY, Naumova AV (2007). Cardiomyocytes derived from human embryonic stem cells in pro-survival factors enhance function of infarcted rat hearts. Nat Biotechnol.

[b2] Mummery C, Ward-van Oostwaard D, Doevendans P (2003). Differentiation of human embryonic stem cells to cardiomyocytes: role of coculture with visceral endoderm-like cells. Circulation.

[b3] Kehat I, Khimovich L, Caspi O (2004). Electromechanical integration of cardiomyocytes derived from human embryonic stem cells. Nat Biotechnol.

[b4] Laake van LW, Passier R, Monshouwer-Kloots J (2007). Human embryonic stem cell-derived cardiomyocytes survive and mature in the mouse heart and transiently improve function after myocardial infarction. Stem Cell Res.

[b5] Muller-Ehmsen J, Whittaker P, Kloner RA (2002). Survival and development of neonatal rat cardiomyocytes transplanted into adult myocardium. J Mol Cell Cardiol.

[b6] Teng CJ, Luo J, Chiu RC (2006). Massive mechanical loss of microspheres with direct intramyocardial injection in the beating heart: implications for cellular cardiomyoplasty. J Thorac Cardiovasc Surg.

[b7] Hou D, Youssef EA, Brinton TJ (2005). Radiolabeled cell distribution after intramyocardial, intracoronary, and interstitial retrograde coronary venous delivery: implications for current clinical trials. Circulation.

[b8] Hudson W, Collins MC, deFreitas D (2007). Beating and arrested intramyocardial injections are associated with significant mechanical loss: implications for cardiac cell transplantation. J Surg Res.

[b9] Zhang M, Methot D, Poppa V (2001). Cardiomyocyte grafting for cardiac repair: graft cell death and anti-death strategies. J Mol Cell Cardiol.

[b10] Mummery CL, Zhang J, Ng ES (2012). Differentiation of human embryonic stem cells and induced pluripotent stem cells to cardiomyocytes: a methods overview. Circ Res.

[b11] Zhu WZ, Biber Van B, Laflamme MA (2011). Methods for the derivation and use of cardiomyocytes from human pluripotent stem cells. Methods Mol Biol.

[b12] Pfannkuche K, Neuss S, Pillekamp F (2010). Fibroblasts facilitate the engraftment of embryonic stem cell-derived cardiomyocytes on three-dimensional collagen matrices and aggregation in hanging drops. Stem Cells Dev.

[b13] Laake van LW, Donselaar van EG, Monshouwer-Kloots J (2010). Extracellular matrix formation after transplantation of human embryonic stem cell-derived cardiomyocytes. Cell Mol Life Sci.

[b14] Ng SY, Wong CK, Tsang SY (2010). Differential gene expressions in atrial and ventricular myocytes: insights into the road of applying embryonic stem cell-derived cardiomyocytes for future therapies. Am J Physiol Cell Physiol.

[b15] Robey TE, Saiget MK, Reinecke H (2008). Systems approaches to preventing transplanted cell death in cardiac repair. J Mol Cell Cardiol.

[b16] Han Y, Kuang SZ, Gomer A (2010). Hypoxia influences the vascular expansion and differentiation of embryonic stem cell cultures through the temporal expression of vascular endothelial growth factor receptors in an ARNT-dependent manner. Stem Cells.

[b17] O'Rourke B (2004). Evidence for mitochondrial K^+^ channels and their role in cardioprotection. Circ Res.

[b18] Niagara MI, Haider H, Jiang S (2007). Pharmacologically preconditioned skeletal myoblasts are resistant to oxidative stress and promote angiomyogenesis *via* release of paracrine factors in the infarcted heart. Circ Res.

[b19] Shintani S, Kusano K, Ii M (2006). Synergistic effect of combined intramyocardial CD34^+^ cells and VEGF2 gene therapy after MI. Nat Clin Pract Cardiovasc Med.

[b20] Azarnoush K, Maurel A, Sebbah L (2005). Enhancement of the functional benefits of skeletal myoblast transplantation by means of coadministration of hypoxia-inducible factor 1alpha. J Thorac Cardiovasc Surg.

[b21] Tang YL, Zhu W, Cheng M (2009). Hypoxic preconditioning enhances the benefit of cardiac progenitor cell therapy for treatment of myocardial infarction by inducing CXCR4 expression. Circ Res.

[b22] Braam SR, Nauw R, Ward-van Oostwaard D (2010). Inhibition of ROCK improves survival of human embryonic stem cell-derived cardiomyocytes after dissociation. Ann N Y Acad Sci.

[b23] Singla DK, Singla RD, Lamm S (2011). TGF-beta2 treatment enhances cytoprotective factors released from embryonic stem cells and inhibits apoptosis in infarcted myocardium. Am J Physiol Heart Circ Physiol.

[b24] Yeghiazarians Y, Gaur M, Zhang Y (2012). Myocardial improvement with human embryonic stem cell-derived cardiomyocytes enriched by p38MAPK inhibition. Cytotherapy.

[b25] Yin Q, Jin P, Liu X (2011). SDF-1alpha inhibits hypoxia and serum deprivation-induced apoptosis in mesenchymal stem cells through PI3K/Akt and ERK1/2 signaling pathways. Mol Biol Rep.

[b26] Kutschka I, Kofidis T, Chen IY (2006). Adenoviral human BCL-2 transgene expression attenuates early donor cell death after cardiomyoblast transplantation into ischemic rat hearts. Circulation.

[b27] Maekawa Y, Mizue N, Chan A (2009). Survival and cardiac remodeling after myocardial infarction are critically dependent on the host innate immune interleukin-1 receptor-associated kinase-4 signaling: a regulator of bone marrow-derived dendritic cells. Circulation.

[b28] Murtuza B, Suzuki K, Bou-Gharios G (2004). Transplantation of skeletal myoblasts secreting an IL-1 inhibitor modulates adverse remodeling in infarcted murine myocardium. Proc Natl Acad Sci USA.

[b29] Suzuki K, Murtuza B, Beauchamp JR (2004). Dynamics and mediators of acute graft attrition after myoblast transplantation to the heart. FASEB J.

[b30] Nussbaum J, Minami E, Laflamme MA (2007). Transplantation of undifferentiated murine embryonic stem cells in the heart: teratoma formation and immune response. FASEB J.

[b31] Hodgetts SI, Beilharz MW, Scalzo AA (2000). Why do cultured transplanted myoblasts die *in vivo*? DNA quantification shows enhanced survival of donor male myoblasts in host mice depleted of CD4^+^ and CD8^+^ cells or Nk1.1^+^ cells. Cell Transplant.

[b32] Murakami M, Ito H, Harada E (2006). Long-term survival of xenogeneic heart grafts achieved by costimulatory blockade and transient mixed chimerism. Transplantation.

[b33] Goh G, Self T, Barbadillo Munoz MD (2005). Molecular and phenotypic analyses of human embryonic stem cell-derived cardiomyocytes: opportunities and challenges for clinical translation. Thromb Haemost.

[b34] Retuerto MA, Schalch P, Patejunas G (2004). Angiogenic pretreatment improves the efficacy of cellular cardiomyoplasty performed with fetal cardiomyocyte implantation. J Thorac Cardiovasc Surg.

[b35] Sakakibara Y, Nishimura K, Tambara K (2002). Prevascularization with gelatin microspheres containing basic fibroblast growth factor enhances the benefits of cardiomyocyte transplantation. J Thorac Cardiovasc Surg.

[b36] Christman KL, Vardanian AJ, Fang Q (2004). Injectable fibrin scaffold improves cell transplant survival, reduces infarct expansion, and induces neovasculature formation in ischemic myocardium. J Am Coll Cardiol.

[b37] Dai W, Wold LE, Dow JS (2005). Thickening of the infarcted wall by collagen injection improves left ventricular function in rats: a novel approach to preserve cardiac function after myocardial infarction. J Am Coll Cardiol.

[b38] Garbern JC, Minami E, Stayton PS (2011). Delivery of basic fibroblast growth factor with a pH-responsive, injectable hydrogel to improve angiogenesis in infarcted myocardium. Biomaterials.

[b39] Kofidis T, Bruin de JL, Hoyt G (2005). Myocardial restoration with embryonic stem cell bioartificial tissue transplantation. J Heart Lung Transplant.

[b40] Kutschka I, Chen IY, Kofidis T (2006). Collagen matrices enhance survival of transplanted cardiomyoblasts and contribute to functional improvement of ischemic rat hearts. Circulation.

[b41] Landa N, Miller L, Feinberg MS (2008). Effect of injectable alginate implant on cardiac remodeling and function after recent and old infarcts in rat. Circulation.

[b42] Lu S, Wang H, Lu W (2010). Both the transplantation of somatic cell nuclear transfer- and fertilization-derived mouse embryonic stem cells with temperature-responsive chitosan hydrogel improve myocardial performance in infarcted rat hearts. Tissue Eng Part A.

[b43] Meng Y, Eshghi S, Li YJ (2010). Characterization of integrin engagement during defined human embryonic stem cell culture. FASEB J.

[b44] Ou L, Li W, Zhang Y (2011). Intracardiac injection of matrigel induces stem cell recruitment and improves cardiac functions in a rat myocardial infarction model. J Cell Mol Med.

[b45] Ye Z, Zhou Y, Cai H (2011). Myocardial regeneration: roles of stem cells and hydrogels. Adv Drug Deliv Rev.

[b46] Yoon SJ, Fang YH, Lim CH (2009). Regeneration of ischemic heart using hyaluronic acid-based injectable hydrogel. J Biomed Mater Res B Appl Biomater.

[b47] Cheng K, Shen D, Smith J (2012). Transplantation of platelet gel spiked with cardiosphere-derived cells boosts structural and functional benefits relative to gel transplantation alone in rats with myocardial infarction. Biomaterials.

[b48] Smith RR, Marban E, Marban L (2013). Enhancing retention and efficacy of cardiosphere-derived cells administered after myocardial infarction using a hyaluronan-gelatin hydrogel. Biomatter.

[b49] Yoon DM, Curtiss S, Reddi AH (2009). Addition of hyaluronic acid to alginate embedded chondrocytes interferes with insulin-like growth factor-1 signaling *in vitro* and *in vivo*. Tissue Eng Part A.

[b50] Cheng K, Blusztajn A, Shen D (2012). Functional performance of human cardiosphere-derived cells delivered in an *in situ* polymerizable hyaluronan-gelatin hydrogel. Biomaterials.

[b51] Zhang G, Nakamura Y, Wang X (2007). Controlled release of stromal cell-derived factor-1 alpha *in situ* increases c-kit+ cell homing to the infarcted heart. Tissue Eng.

[b52] Wang T, Jiang XJ, Lin T (2009). The inhibition of postinfarct ventricle remodeling without polycythaemia following local sustained intramyocardial delivery of erythropoietin within a supramolecular hydrogel. Biomaterials.

[b53] Wang H, Liu Z, Li D (2012). Injectable biodegradable hydrogels for embryonic stem cell transplantation: improved cardiac remodelling and function of myocardial infarction. J Cell Mol Med.

[b54] Cheng K, Li TS, Malliaras K (2010). Magnetic targeting enhances engraftment and functional benefit of iron-labeled cardiosphere-derived cells in myocardial infarction. Circ Res.

[b55] Penn MS (2009). Importance of the SDF-1:CXCR4 axis in myocardial repair. Circ Res.

[b56] Tulloch NL, Muskheli V, Razumova MV (2011). Growth of engineered human myocardium with mechanical loading and vascular coculture. Circ Res.

[b57] Narita T, Shintani Y, Ikebe C (2013). The use of scaffold-free cell sheet technique to refine mesenchymal stromal cell-based therapy for heart failure. Mol Ther.

